# Investigating the effects of effective stress on pore-dependent permeability measurements of crushed coal

**DOI:** 10.1371/journal.pone.0261678

**Published:** 2021-12-23

**Authors:** Mingkun Pang, Tianjun Zhang, Lu Gao, Binfeng Qin

**Affiliations:** 1 College of Safety Science and Engineering, Xi’an University of Science and Technology, Xi’an, China; 2 Key Laboratory of Western Mine Exploitation and Hazard Prevention of the Ministry of Education, Xi’an, China; University of Nottingham, UNITED KINGDOM

## Abstract

The accurate determination of permeability is one of the parameters essential for the study of fluid flow and transport state. However, a large number of fractured coal bodies are faced during the production of coal mines. The study of permeability of these special media composed of grains of a certain size, whose structure is different from that of raw coal, has been in the exploratory stage. In this paper, inspired by the calculation method of median particle size and the calculation principle of KC’s equation, we calculate the permeability parameters of broken coal particles. It is considered that its permeability is closely related to the compaction and re-crushing process of skeletal grains. The lateral limit compression test of the crushed coal body was designed, and the pore-dominated permeability calculation method was given to reveal the mechanism of the action of the effective stress. The dependence relationship between the effective stress and the pore-correlation permeability is obtained by data analysis, and the force and deformation process of the crushed coal grain media is described. In contrast to the conventional Darcy series of permeability discussions, our approach excludes the influence of fluid factors on the permeability of porous media. The permeability of porous media is considered to be determined only by its own pore structure.

## Introduction

The permeability is a quantity used to quantitatively characterize the ease of fluid passage in porous media [1]. The magnitude of its value directly affects the fluid transport process and fluid pressure. Many theoretical models have been proposed to accurately determine the permeability of fractured coal bodies [2, 3]. In 1856, Darcy summarized the famous Darcy’s law of permeability of porous media based on water permeation tests in a sand column. In 1937, Kozeny and Carman improved the calculation of Darcy’s permeability k and proposed the KC’s equation [4, 5]. Bear and Costa considered Kozeny-Carman (KC) equation is the simplest equation and is applicable to porous media materials [6, 7].

$$K=\frac{\phi^{3}}{C{\left(1-\phi \right)}^{2}S^{2}}$$ (1)

Where, the *C* denotes the Kozeny constant. The *S* denotes the specific surface area. However, the *C* is an empirical parameter who is related to the initial porosity of the medium. For the Kozeny constant of the crushed coal body medium, Carman has demonstrated through extensive experiments that its calculated value is about 5 [8]. We found that the study of permeability of fractured coal bodies is essentially an investigation of the permeability properties of a medium with variable porosity. At the present stage, the main methods for the experimental determination of the permeability characteristics of fractured coal bodies include: the steady-state permeability method and the transient permeability method [9, 10]. Hydraulic conductivity characteristics are: the flow rate through a unit cross-sectional area per unit hydraulic gradient, and its value depends mainly on the composition and structure of the porous medium, and also on the fluid properties [11]. For porous media with low permeability, Dewers et al. [12] showed that the permeability of crushed coals can vary by several orders of magnitude at different effective stresses. The measured data of Dong et al. and Drews et al. showed [13, 14] that for Barnett, Muskwa and Ohio fractured specimens, the permeability is exponentially related to the effective stress. In hydrogeology, Katsuki et al. [15] tested the permeability of standard coal bodies using steady-state permeability method and pulse decay method, giving the permeability of shale reservoirs will be exponentially decreasing with the increase of effective stress. Due to the difficulty of core sampling, Cso´ka´s proposed an integrated interpretation method, which is based purely on logging methods to calculate the permeability and hydraulic conductivity of water formations [16]. However, for some phenomena, such as this calculation of permeability due to pore-related structures, it is a difficult problem to determine the permeability of fractured coal bodies accurately.

In this paper, using the calculation principle of KC’s equation and drawing on the calculation method of median particle size, the permeability parameters of different pore structures are calculated and analyzed, and the details of how to correctly interpret the calculation results of stress-related permeability with the effective stress formula are presented. The infiltration channels are also further analyzed to give the damage characteristics of such a system composed of broken coal bodies in the crushed coal medium, including the infiltration characteristics of the skeleton and the compaction characteristics of the particles.

### Pore-dependent permeability

The permeability of this medium composed of coal particles is mainly determined by the pore size, so for the study of the porosity of this medium, it mainly focuses on the determination of the calculation method of permeability.

### Definition of pore-correlation permeability

Permeability is a fundamental parameter describing the strength of the ability to allow fluid passage within a porous medium, which is related only to the skeletal structure and not to the fluid properties [17, 18]. The well-known Kozeny-Carman (KC) equation, which is the most classical formulation of the permeability-porosity relationship, is widely used in the study of seepage problems in porous media in engineering, and it is the most fundamental formula for many permeability model studies. The most important contribution of the KC’s equation is that it directly relates the permeability K to the porosity A [19, 20]. The most important contribution of the KC’s equation is the direct correlation between permeability *K* and porosity *ϕ*. However, in the process of calculating the permeability of the crushed coal rock body using the KC’s equation, the specific surface area of the crushed coal rock body is difficult to calculate, so we make the following transformations. Suppose, the crushed coal body grains are considered as spheres of unequal diameter with radius *r*_i_ and the number is N. The *V*_b_ stands for the sum of solid grain volume and pore volume. *ϕ* stands for the porosity of the crushed coal body. Then the total surface area of pores $A_{s}=\sum_{i}^{N}4\pi r_{i}^{2}$ and the total volume of solid particles $S=A_{S}/V_{b}=\frac{3\left(1-\phi \right)}{r}$. The specific surface area $V_{s}=\sum_{i}^{N}\frac{4}{3}\pi r_{i}^{3}$. At this point, we take spherical grains as the subject of study, so *d* = 2*r* [21, 22].

$$K=\frac{\phi^{3}}{36C{\left(1-\phi \right)}^{2}}d^{2}$$ (2)

Where: *d* is the average diameter of spherical particles, Liu et al. [23] whose value can be taken as *d*_50_ from the grading curve.

### Pore-correlation permeability model

Although the KC’s equation is widely accepted and used, it has had many limitations since its inception. In essence, the equation is a semi-empirical relation, and it has been shown that the KC’s constant is not a constant, it is a quantity related to porosity [24]. The KC’s model is frequently modified and has different modified versions to improve the estimation of permeability. Some common forms of KC’s equations are given in the table below. By studying the pore structure of textiles, McGregor [25] used the KC’s equation as a theoretical basis to simulate the flow of dye in textile yarn packages. Bourbie´s et al. [26] proposed a special repair method using a variable power function for porosity, which is also applicable to low porosity, with n taking values ranging from the derived value of 3 for large porosity to the derived value for very low porosity, about between 7 and 8. In addition, the correction of the KC’s equation by considering the effective porosity and percolation rate [28, 29], Archie’s law [30–32] is also used for the derivation of permeability. The KC’s equations for different porous media are shown in Table 1.

**Table 1:** KC’s equation and its modifications for different porous media.

Reference	Permeability equation	Applicable medium
McGregor [25]	$K=\frac{d^{2}}{16c}\frac{\phi^{3}}{{\left(1-\phi \right)}^{2}}$	Textile assembly
Bourbie´ et al. [26]	*K* = *Cϕ*^*n*^*d*^2^	Porous media
Rodriguez et al. [27]	$K=\frac{\phi^{n+1}}{C{\left(1-\phi^{2}\right)}^{n}}$	Glass and fiber
Mavko and Nur [28]	*K* = *Cd*^2^(*ϕ-ϕ*_*c*_)^3^/(1+*ϕ*_*c*_*+ϕ*)^2^	Sanstone carbonate
Bayles et al. [29]	$K=C\frac{\phi^{2+n}}{{\left(1-\phi \right)}^{2}}$	Fine particle filter cakes
Costa [30]	*K* = *Cϕ*^*n*^*/1-ϕ*	Fiber mats vesicular rocks

Note: *C* is permeability factor, n is empirical exponent, *ϕ*_*c*_ is percolation threshold.

### Experiments

In order to study the change of permeability of crushed coal grains during compression, we carried out the "lateral-limit compression test" on crushed coal grains. We prepared specimens with different values of n, because the Talbol’s index n can reflect the compactness of the mixed material. The greater the compactness of the mixed material, the smaller the porosity, so the Talbol’s index n also indirectly reflects the porosity of the mixed material. Based on this, we prepared specimens containing different initial porosity using the Talbol’s power theory, and changed their compactness state by controlling their stress loading state to analyze the variation law of their permeability.

### Materials

#### Research background

YANJIAHE coal mine is located in Xunyi County, northwest of Xi’an City, Shaanxi Province, China, and belongs to the southwest of Longdong Loess Plateau, the coal-bearing stratum is Middle Jurassic Yan’an Group (J2y), 8 coal is the main mineable coal seam in the area, 8 coal seam in the area drilling reveals burial depth 101.25 ~ 701.62m, average 509.37m. 8 coal seam dry state compressive strength *R*_b_ = 27.45 MPa, saturation compressive strength *R*_w_ = 10.54 MPa, softening coefficient *μ* = 0.38, natural tensile strength *R*_t_ = 0.87 MPa.

#### Material preparation

Considering that the lateral deformation of the crushed specimens in the permeameter is constrained, so the deformation and compression density of the specimens are controllable, we hold the view that the change of pore structure is the main factor affecting the change of permeability of crushed coal grains. Therefore, coal particles of different particle diameters were used, including four particle sizes 0.5 mm, 1.0 mm, 2.5 mm, and 5.0 mm. considering the volume of the test cylinder and the volume compression range of the samples, the total mass of each group of samples was selected as 500 g. Then the Talbol’s grading theory was used to calculate the mass ratio of each particle size range, and the mass ratio of each particle size range was calculated according to the Talbol’s grading theory. The mass ratio of each particle size range was calculated by using Talbol’s grading theory, and the samples were numbered from sample 1, sample 2, to sample 10 in the order of Talbol’s power index n. The mass ratio of each particle size range is shown in Table 2.

**Table 2:** The ratio of particle size in the sample.

*n*	0.1	0.2	0.3	0.4	0.5	0.6	0.7	0.8	0.9
0.5	397.2	315.5	250.6	199.1	158.1	125.6	99.8	79.2	62.9
1.0	28.5	46.9	57.9	63.6	65.5	64.8	62.3	58.7	54.5
2.5	40.8	72.9	97.6	116.3	129.9	139.5	145.7	149.2	150.5
5.0	33.5	64.7	93.9	121.1	146.4	170.1	192.2	212.8	232.1

#### Experimental procedure

The test process is divided into the following important steps: 1) Preparation of crushed coal samples. The crushed coal particles used in this test were taken from Yanjiahe coal mine in Shaanxi province, and each block taken back was crushed, and four particle sizes were screened out using a vibrating sieve. 2) Preparation of gradation structure specimens. The crushed coal particles of different particle sizes were prepared according to the Talbol’s formula, and the values of Talbol’s power index n were taken from 0.1 to 1.0.

$$P_{i}={\left(d_{i}/D\right)}^{n}\times 100\text{\%}$$ (3)

Where: *P*_*i*_ indicates the mass proportion of the largest diameter grain to be calculated; *n* is the Talbol’s index parameter, the larger the value of *n* characterizes the proportion of large grains in the specimen. *D* is the largest grain size in the broken coal specimen. *d*_*i*_ indicates the grain diameter to be calculated.

**Fig 1:**
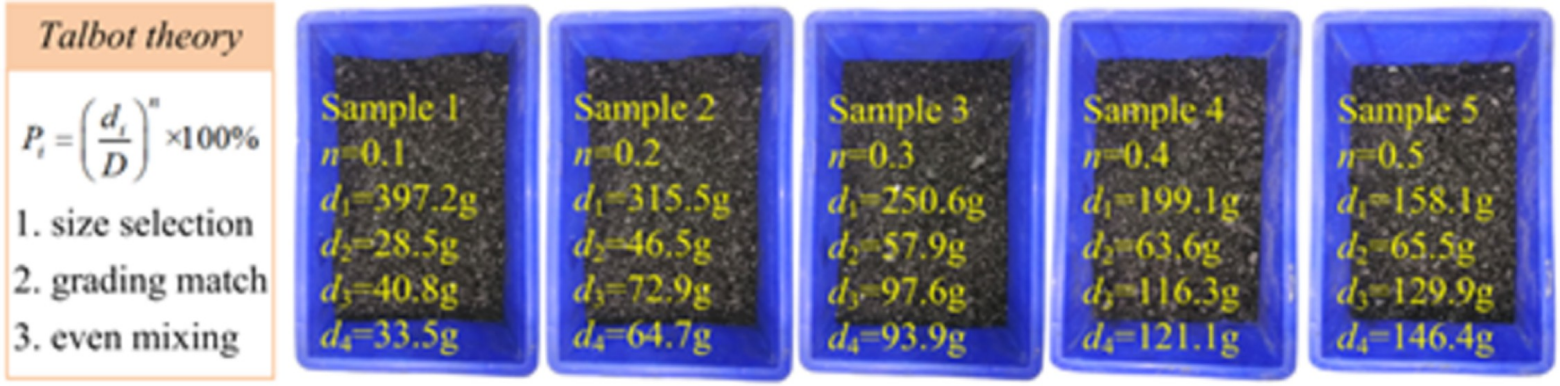
Sample preparation process.

3) Assembly of crushed specimens. The specimens were loaded into the cylinder in the order of coded specimens, and the permeation tests were performed one by one.4) Experimental testing. The specimens are carried out one by one according to the preset axial pressure gradient, permeation pressure gradient and different gradation structure specimens, and the data are recorded. The specimen preparation process is shown in Fig 1.

In the experiments, five stages of axial loading of 1, 5, 10, 20 and 30 kN were used to perform progressive loading seepage tests on coal specimens with different grading structures. Each axial loading test was carried out at seepage pressure levels of 0.5, 1.0, 1.5, 2.0, and 2.5 MPa, respectively, and the axial loading F, axial displacement *Δ*h, and loading time t were recorded by the computer system.

### Methodologies

#### Experiment design

**Fig 2:**
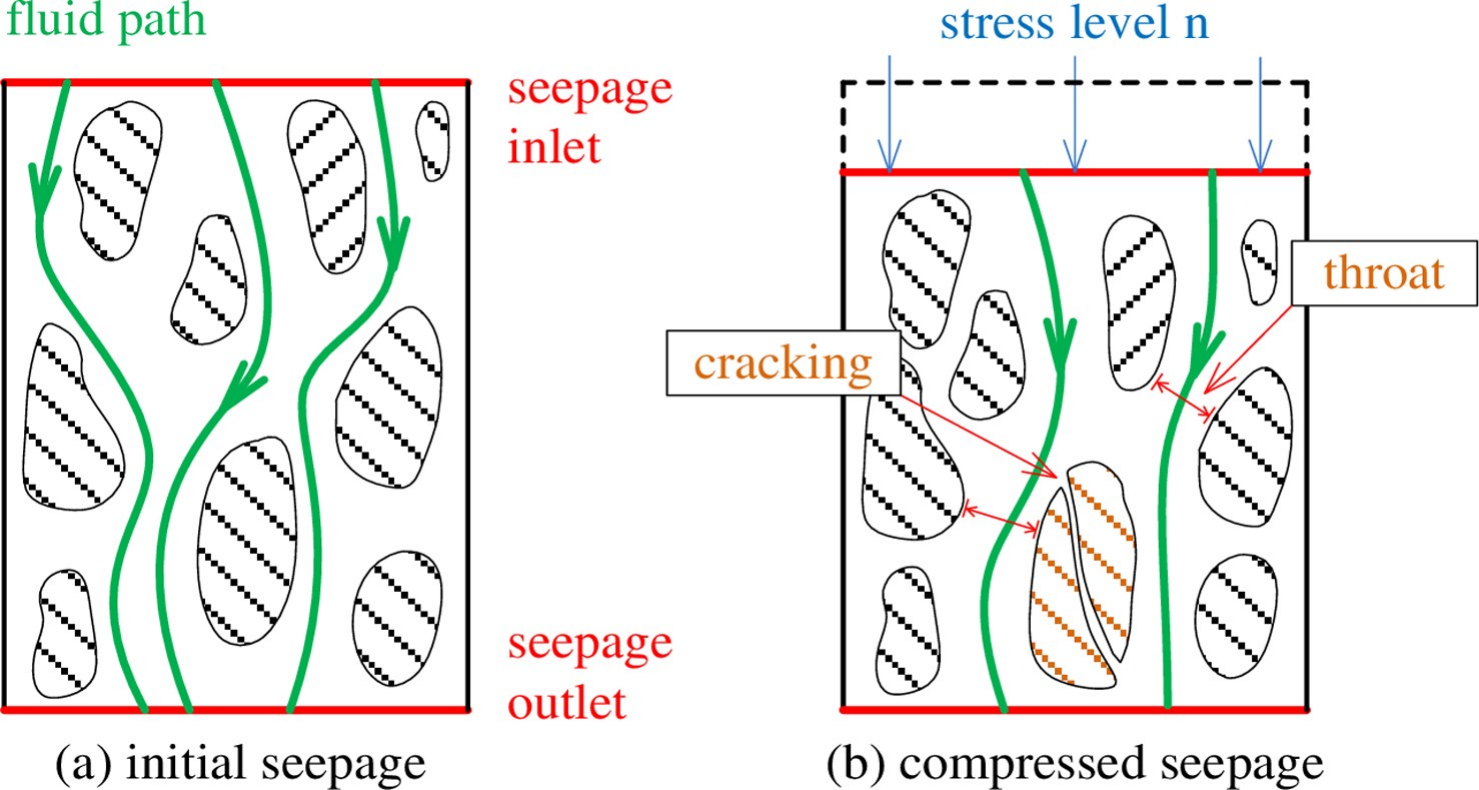
Structural deformation diagram.

The permeability characteristics of fractured rocks are closely related to their porosity, and the study of Talbol’s index n is essential to study the permeability characteristics of fractured rocks. According to the experimental design, the percolation tests of coal with different graded structures were carried out under graded loading, with 5 levels of loading applied axially to provide 5 levels of stress conditions, which were noted as stress 1, stress 2, …, stress 5. Five levels of penetration pressure are set for each axial load, providing 10 levels of penetration pressure, which are noted as seepage 1, seepage 2, … …, seepage 5. A schematic diagram of the structural deformation of the crushed specimen is shown in Fig 2.

#### Experimental equipment

**Fig 3:**
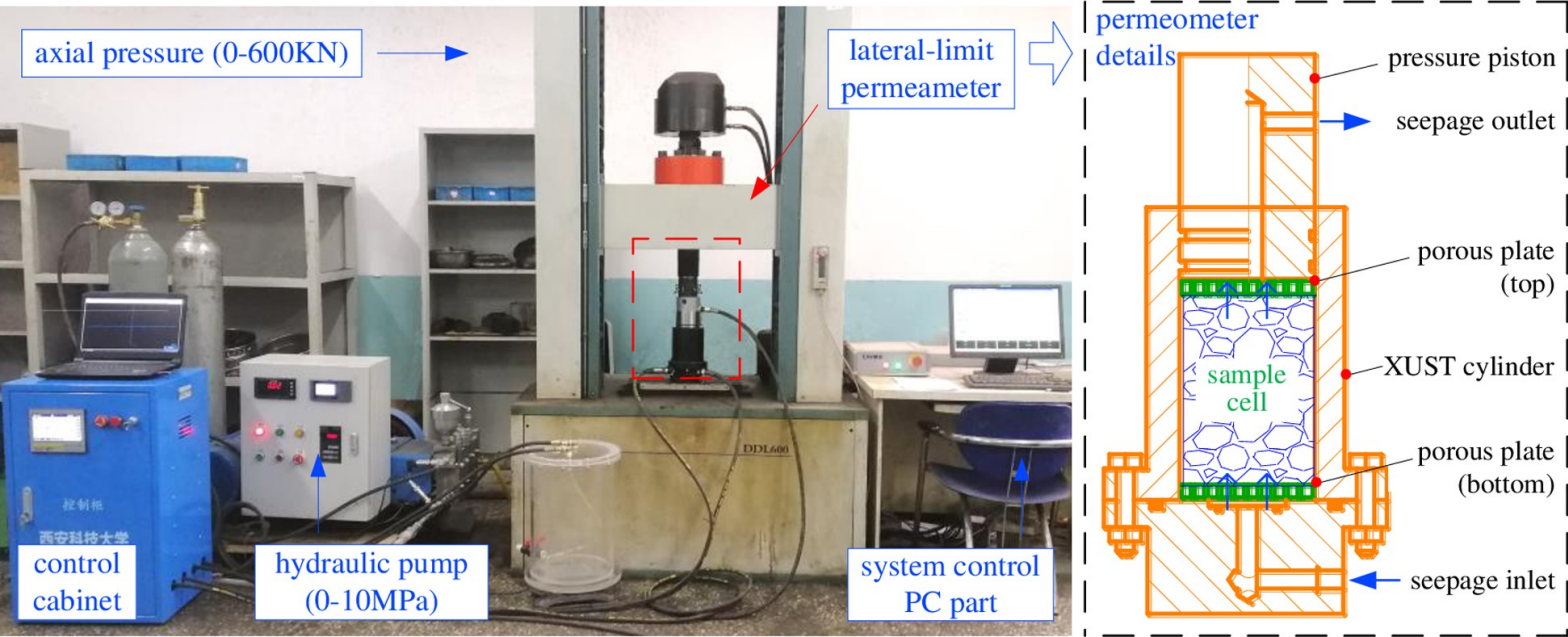
Sample installation diagram.

In order to provide such special seepage mechanics conditions, a lateral limit compression seepage test system (XUST001) was homemade, mainly including: axial pressure control part, permeation pressure control part, and data acquisition part; the system can realize axial pressure range (0-600KN), seepage pressure range (0-10MPa), etc. The schematic diagram of specimen installation is shown in Fig 3.

## Results and discussions

The variation of the pore structure of the crushed coal body grains determines its permeability characteristics. Therefore, the authors will start from the pore permeability model of the crushed coal medium, calculate the corresponding permeability parameters, and analyze the influence of several factors, such as permeability behavior, evolution history, porosity and effective stress, on the pore-dependent permeability of the coal body.

### Calculation of pore-dependent permeability parameters

**Fig 4:**
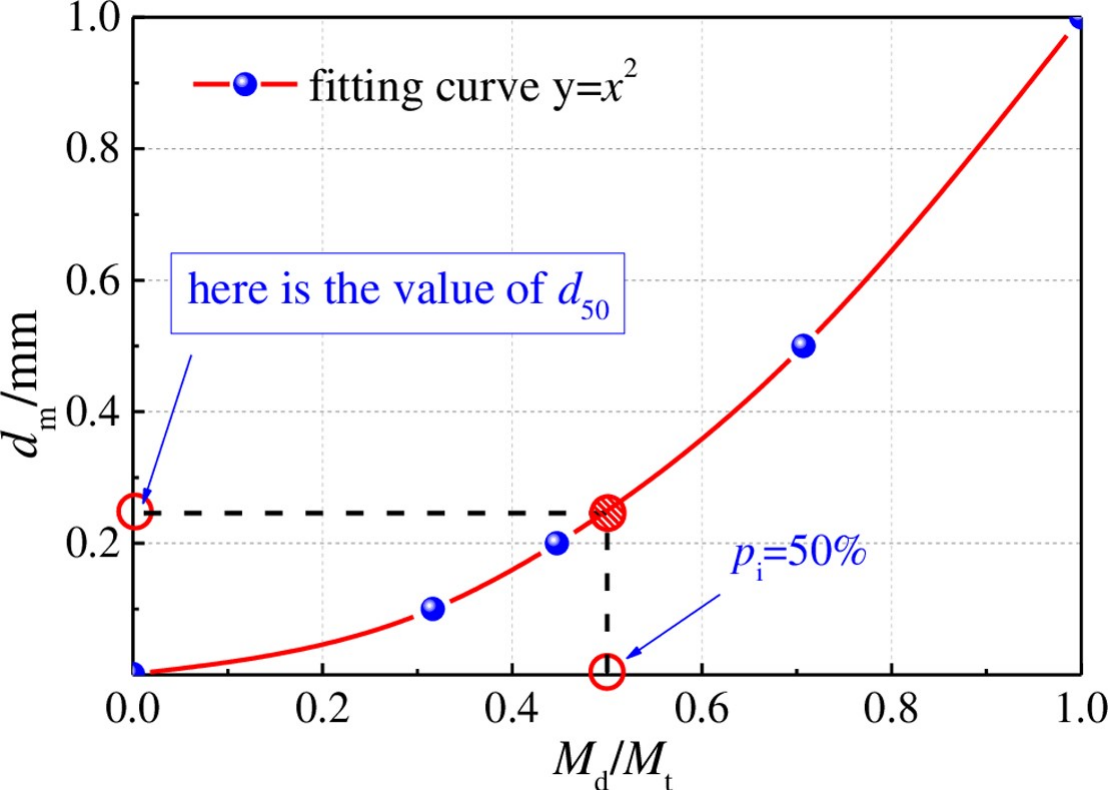
Principle of calculating the *d*_50_ value of the specimen.

The Particle size-average is a size value in the selected sample, the number of particles larger than this size value accounts for 50% and the number of particles smaller than this size value also accounts for 50%, it is called this size value as the particle size-average value. It is generally considered that the crushed coal grains belong to spheres of unequal diameter, and its average diameter can be obtained from this curve (*d*_50_). The calculation principle of the value of *d*_50_ is shown in Fig 4.

During the calculation, several parameters must be calculated, *M*_d_/*M*_t_ indicates the ratio of the mass of that particle size to the total mass; *d*_m_ indicates the ratio of that particle size to the maximum particle size. The calculated values of *d*_50_ were obtained for all 10 groups of specimens by bringing in the corresponding specimen parameters. the *d*_50_ calculation table is shown in Table 3.

**Table 3:** Calculation table of *d*_50_ values.

n	0.1	0.2	0.3	0.4	0.5	0.6	0.7	0.8	0.9
coefficient	10.0	5.0	3.3	2.5	2.0	1.7	1.4	1.3	1.1
*d*_50_	0.005	0.156	0.497	0.884	1.250	1.576	1.869	2.102	2.315

### Evolutionary history of pore-dependent permeability

**Fig 5:**
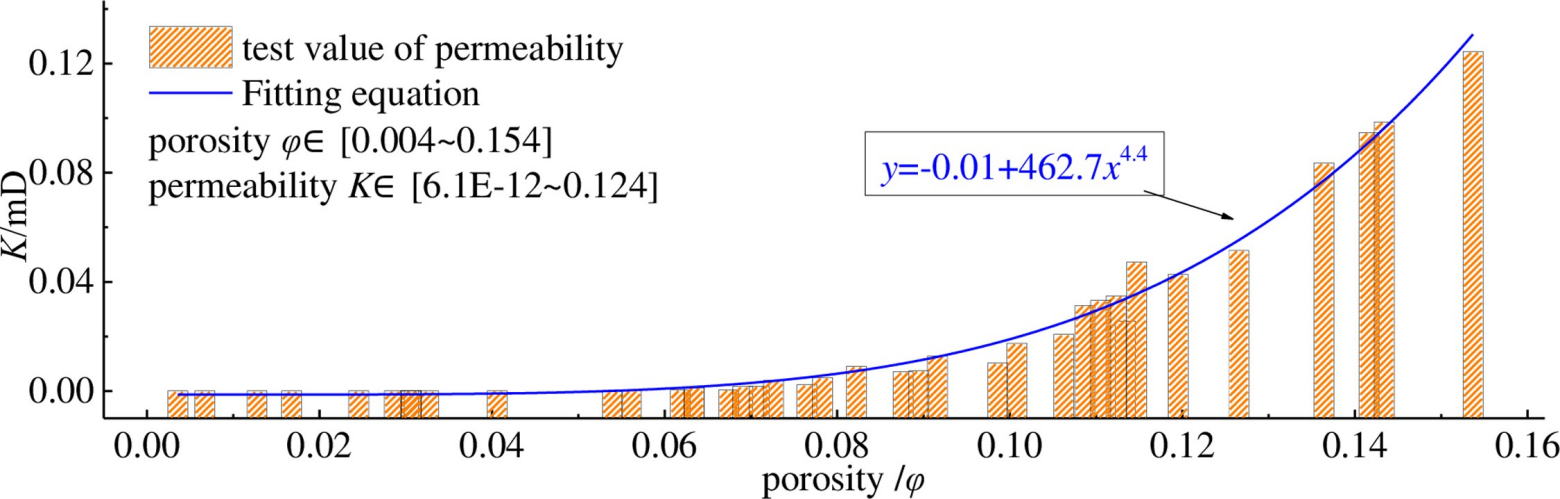
History of pore-associated permeability values.

The pore characteristics of the crushed coal medium determine the permeability of the coal body, while the pore and fracture characteristics of the coal particles mainly depend on the coal rock composition and coal grade. For this permeable medium composed of crushed coal particles, an important part of its study is the damage characteristics of the pore system, including the permeability characteristics of the skeleton and the compaction characteristics of the particles. The history of pore-dependent permeability values is shown in Fig 5.

**Fig 6:**
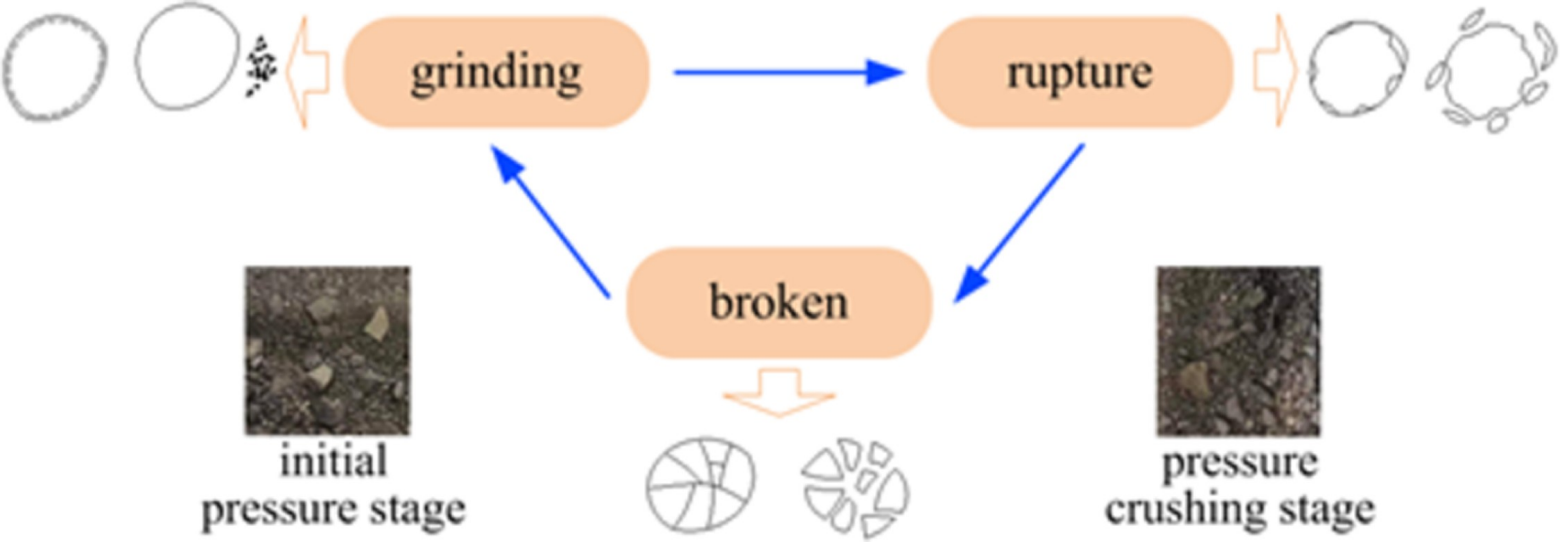
Structural evolution process of broken coal particles.

From the figure, we can get that in the whole permeation process, the specimens with different initial porosity, under the action of different axial pressure, their porosity obeys a similar change law, the porosity at all levels gradually decreases, while the permeability gradually increases. The structural evolution process of broken coal particles is shown in Fig 6.

The essential reason is that the deformation of the pore structure of this crushed coal medium determines the change of its permeability characteristics, and the change of axial displacement in the compaction process must cause the change of porosity, thus causing the decrease of permeability rate. Under high axial stress conditions, further grain rupture will occur within the already dense crushed coal specimens. It will cause the pore channels inside the specimen to close and the flow rate to change abruptly. Under the action of high pore pressure, it may cause the movement of large particles inside the porous medium, and even the grinding of some small particles, when the permeable skeleton is extremely unstable. A sharp increase in the porosity parameter can lead to an increase in flow velocity and the conversion of percolation into pipe flow.

### Determination of porosity on pore—dependent permeability

**Fig 7:**
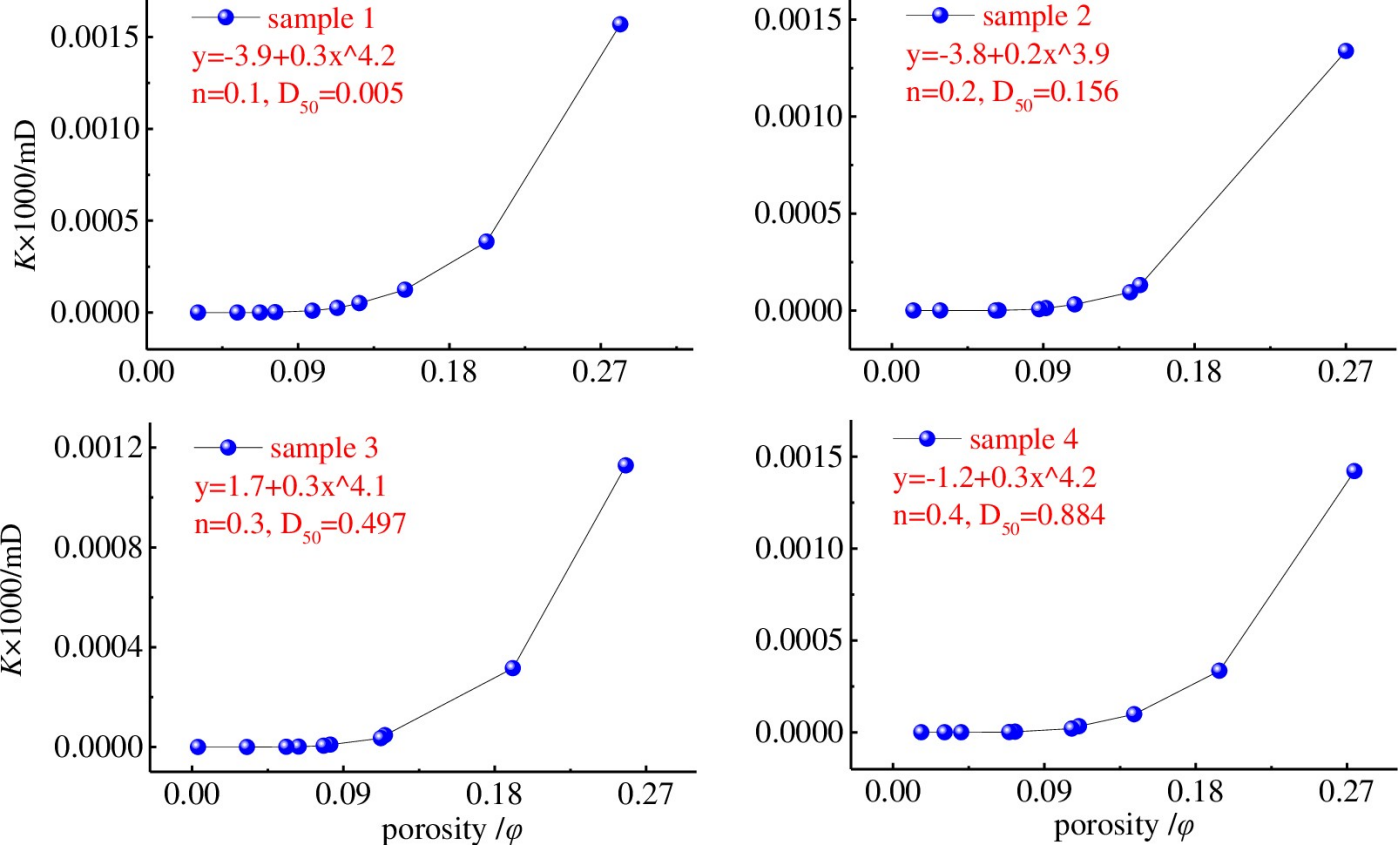
Relationship between effective stress and permeability of samples.

In general, after the coal specimen is loaded, the porous media skeleton is deformed, and the pore structure may increase or may show a slight increase process, and these phenomena are closely related to the misalignment, fragmentation and dislodgement of the grains inside the porous media, and depend on the number of effective internal percolation channels. The relationship between the effective stress and permeability of the specimen is shown in Fig 7.

Under high stress conditions, their porosity decreases sharply while there is further crushing of coal particles. Eventually, two possibilities coexist: one possibility, part of the roaring channel inside the specimen is closed and the permeability decreases; the other possibility, the particles inside the specimen readjust their posture and new channels are created.

### Dependence between permeability and effective stress

The effective stress is the most fundamental parameter to describe the mechanical properties in this porous medium. Based on this, a number of experts in the relevant fields have conducted an in-depth investigation on the principle of effective stress action, and in 1923 Terzaghi gave the famous one-dimensional compaction effective stress formula in his study of geotechnical porous media [31]. The effective stress *σ* is given as:

$$\sigma_{o}=\sigma +P_{p}$$ (4)

**Fig 8:**
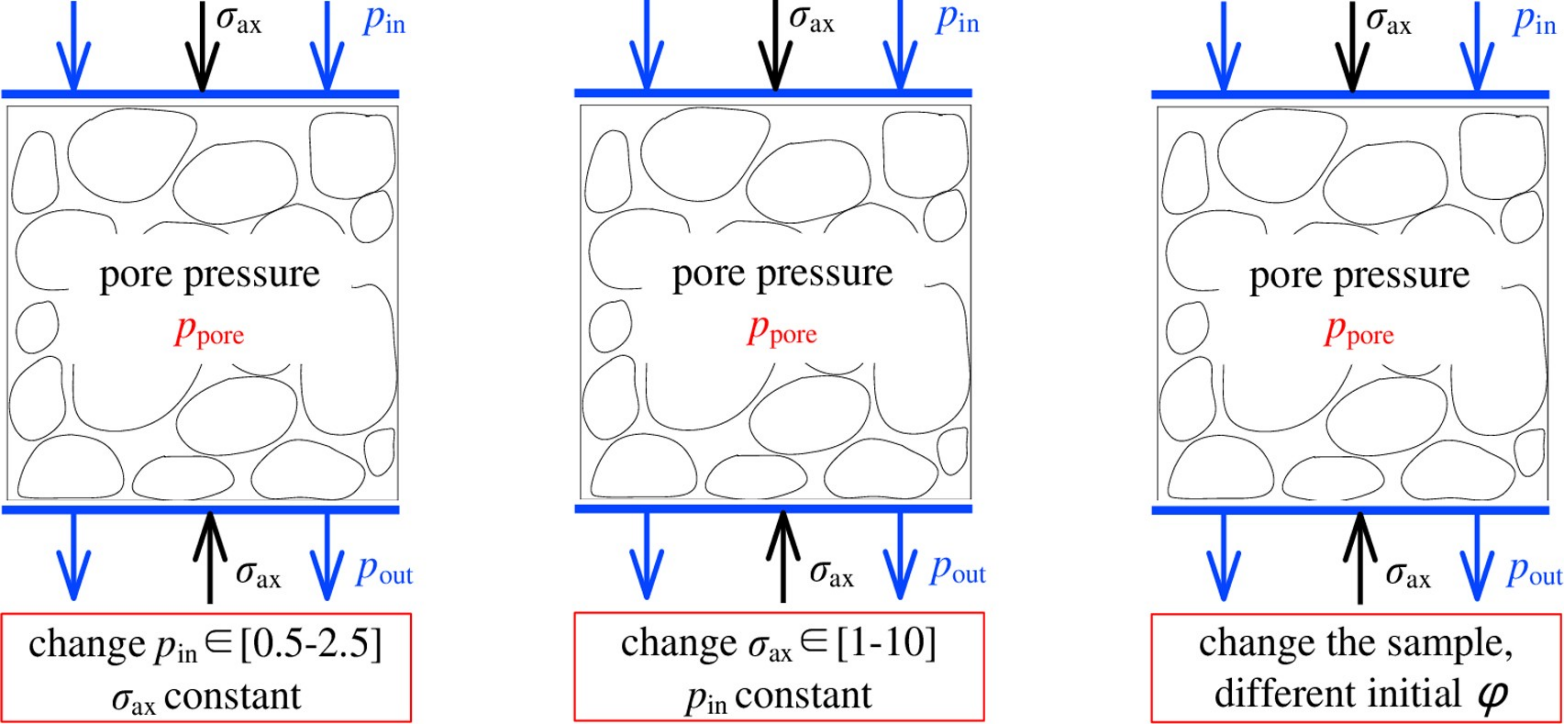
Three different test states of pore structure specimens.

Where: *σ*_*o*_ stands for the stress acting on the whole porous medium, called total stress, MPa; *σ* stands for the stress acting on the solid particles, *P*_*P*_ called effective stress, MPa; it stands for the pressure of the fluid in the pore, called pore pressure, MPa. mainly used in the field of foundation engineering. Three different test states of pore structure specimens are shown in Fig 8.

So far, the continuous improvement of the effective stress calculation formula has been carried out through the joint research of experts in this field. Finally, Heymann et al. [32] explained the stress relationship subjected to porous media considering both skeletal effective stress and structural effective stress and gave an expression for the effective stress true stress.

Effective stress *σ*_*ov*_:

$$\sigma_{ov}\text{=}\left(1-\phi \right)\sigma +\phi P_{p}$$ (5)

**Fig 9:**
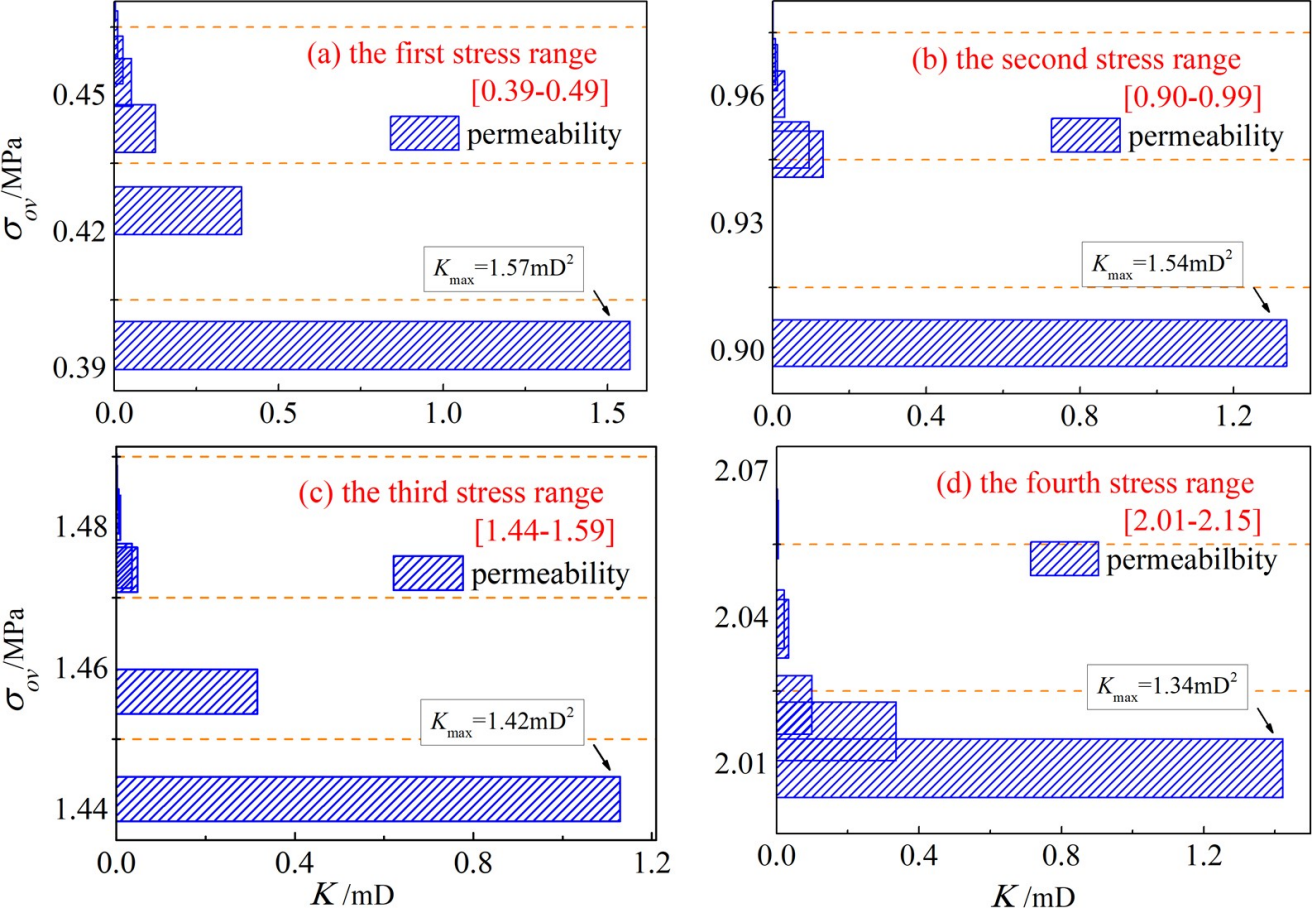
Plot of effective stress versus permeability.

The formula contains the porosity *ϕ*, which is an important parameter to study the percolation of porous media, the formula also analyzes the response relationship between porous media and stress from the perspective of pore structure. A plot of effective stress versus permeability is shown in Fig 9.

Effective stress is a good method, it can analyze the force of the crushed coal particles during the percolation of porous media, which reflects the actual force of coal grains. We analyzed and calculated all the specimens of each group to get the relationship between effective stress and permeability, and the fitted relationship equation is shown in Table 4.

**Table 4:** Fit function and correlation.

sample	*σ*_*ov*_/MPa	formula	*R* ^2^
Alpha -1	0.39–0.49	*y* = exp(21-52*x*)	0.996
Alpha -2	0.90–0.99	*y* = exp(152-58*x*)	0.998
Alpha -3	1.44–1.59	*y* = exp(131-91*x*)	0.997
Alpha -4	2.01–2.15	*y* = exp(388-193*x*)	0.999

Through function fitting, it can be used to characterize the relationship between effective stress and permeability as *y* = exp(*a*-*bx*), and its fitting accuracy is good, which is obtained by normalization: *K* = exp(178–94*σ*_*ov*_). For this fractured medium coal body, the effective stress is the main factor affecting its permeability, and the permeability *k* decreases with the increase of the effective stress *σ*, indicating that the effective stress can cause the closure of some pores inside the coal body and the decrease of the number of seepage channels, which directly leads to the sharp decrease of the permeability of the fractured coal body. The effective stress is the key factor to determine the permeability of the fractured coal rock body. That is, the permeability of the coal rock body for different grading structures decreases exponentially with the increase of the effective stress. In the process of specimen from dense to crushed to re-dense, in the first stage, the skeleton structure of coal rock body is in the fast compressive density stage. In the second stage, the skeleton compressive damage enters the structural readjustment stage.

## Conclusions

The relationship between deformation and damage of broken coal body is discussed through self-designed lateral limit compression test of broken coal body, and the pore-dependent permeability determination method is given to reveal the core factors affecting the permeability change. The main contents include.

1. The law of pore structure change of crushed coal grains is studied, and its essence refers to the process of compaction and re-crushing with the skeleton. Cleverly with the help of KC’s equation and then using the D50 calculation method, and then the permeability parameters of the broken coal grains were determined. Secondly, the deformation and damage characteristics of the crushed coal media system are given by analyzing the permeability channels of the crushed coal body.
2. The accurate determination of the pore-permeability of the crushed coal rock mass is dominated by the effective stress. The pore-correlation permeability calculation method is proposed with the deformation of the pore structure as the main factor of the permeability change of the crushed coal medium. And combined with the measured data of the lateral limit compression test of the crushed coal body, the analysis gives the permeability measurement method considering the pore structure, and further analyzes the evolution history of the permeability to elucidate the mechanism of the effective stress.
3. Effective stress plays a key role in the accurate laboratory determination of pore-correlation permeability. In the laboratory determination of pore-correlation permeability, the permeability of the specimen also decreases with decreasing porosity, in accordance with the relationship: *K* = exp(178–94*σ*_*ov*_). With the increase of penetration pressure, the deformation process of its skeleton can also be divided into three steps, the initial moment shows a rapid decline called rapid pressure density stage. After that, the crushed particles rotate, rub, and attitude adjustment occurs, gradually converging to the best compacted structure, and finally entering the fully compacted state with extremely low porosity.

## Supporting information

S1 Data (ZIP)
